# (2*S*,7*S*)-10-Ethyl-1,8,10,12-tetra­aza­tetra­cyclo­[8.3.1.1^8,12^.0^2,7^]penta­decan-10-ium iodide

**DOI:** 10.1107/S1600536812040159

**Published:** 2012-09-29

**Authors:** Augusto Rivera, Héctor Jairo Osorio, John Sadat-Bernal, Václav Eigner, Michal Dušek

**Affiliations:** a Universidad Nacional de Colombia, Sede Bogotá, Facultad de Ciencias, Departamento de Química, Cra 30 No.45-03, Bogotá, Código Postal 111321, Colombia; b Universidad Nacional de Colombia, Sede Manizales, Colombia; c Department of Solid State Chemistry, Institute of Chemical Technology, Technická 5, 166 28 Prague, Czech Republic; d Institute of Physics ASCR, v.v.i., Na Slovance 2, 182 21 Praha 8, Czech Republic

## Abstract

The title chiral quaternary ammonium salt, C_13_H_25_N_4_^+^·I^−^, was synthesized through the Menschutkin reaction between the cage aminal (2*S*,7*S*)-1,8,10,12-tetra­aza­tetra­cyclo­[8.3.1.1^8,12^.0^2,7^]penta­decane and ethyl iodide. The quaternization occurred regioselectively on the nitrogen with major sp3 character. The crystal structure consists of anions and cations separated by normal distances. Ions are not linked through C—H⋯I hydrogen bonds.

## Related literature

For related structures, see: Becka *et al.* (1963 ▶); Rivera *et al.* (2011*b* ▶,*c* ▶); Rivera, Sadat-Bernal *et al.* (2012 ▶). For the synthesis of the precursor (2*S*,7*S*)-1,8,10,12-tetra­aza­tetra­cyclo [8.3.1.1^8,12^.0^2,7^]penta­decane, see: Rivera, Quiroga *et al.* (2012 ▶). For the preparation of the title salt, see: Rivera *et al.* (2011*a* ▶). For bond-length data, see: Allen *et al.* (1987 ▶). For the structural consequences of the anomeric effect, see: Kakanejadifard & Farnia (1997 ▶); Rivera *et al.* (2011*b* ▶). For synthetic applications of chiral quaternary ammonium salts, see: Lygo Andrews (2004 ▶); Park *et al.* (2004 ▶); Kim & Huh (2001 ▶).
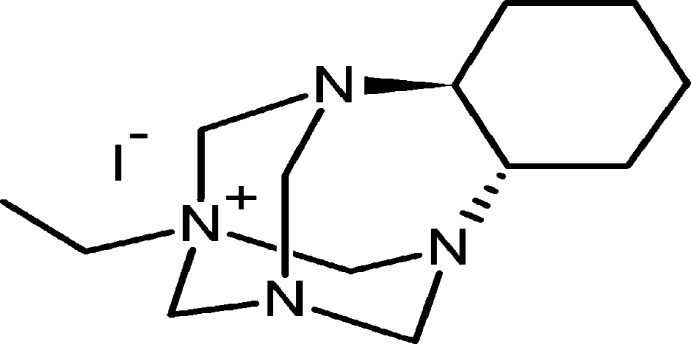


## Experimental

### 

#### Crystal data

C_13_H_25_N_4_^+^·I^−^
*M* *_r_* = 364.3
Orthorhombic, 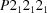

*a* = 10.2227 (5) Å
*b* = 12.0375 (6) Å
*c* = 12.0941 (6) Å
*V* = 1488.25 (13) Å^3^
*Z* = 4
Mo *K*α radiation
μ = 2.14 mm^−1^
*T* = 120 K
0.24 × 0.06 × 0.04 mm

#### Data collection

Agilent Xcalibur (Atlas, Gemini ultra) diffractometer
Absorption correction: multi-scan (*CrysAlis PRO*; Agilent, 2010 ▶) *T*_min_ = 0.746, *T*_max_ = 1
5935 measured reflections
3223 independent reflections
3093 reflections with *I* > 3σ(*I*)
*R*_int_ = 0.016

#### Refinement

*R*[*F* ^2^ > 2σ(*F* ^2^)] = 0.017
*wR*(*F* ^2^) = 0.041
*S* = 1.19
3223 reflections
164 parameters
H-atom parameters constrained
Δρ_max_ = 0.23 e Å^−3^
Δρ_min_ = −0.47 e Å^−3^
Absolute structure: Flack (1983 ▶), 1353 Friedel pairs
Flack parameter: 0.026 (15)

### 

Data collection: *CrysAlis PRO* (Agilent, 2010 ▶); cell refinement: *CrysAlis PRO*; data reduction: *CrysAlis PRO*; program(s) used to solve structure: *Superflip* (Palatinus & Chapuis, 2007 ▶); program(s) used to refine structure: *JANA2006* (Petříček *et al.*, 2006 ▶); molecular graphics: *DIAMOND* (Brandenburg & Putz, 2005 ▶); software used to prepare material for publication: *JANA2006*.

## Supplementary Material

Crystal structure: contains datablock(s) global, I. DOI: 10.1107/S1600536812040159/bh2451sup1.cif

Structure factors: contains datablock(s) I. DOI: 10.1107/S1600536812040159/bh2451Isup2.hkl

Supplementary material file. DOI: 10.1107/S1600536812040159/bh2451Isup3.cml

Additional supplementary materials:  crystallographic information; 3D view; checkCIF report

## Figures and Tables

**Fig. 1.**
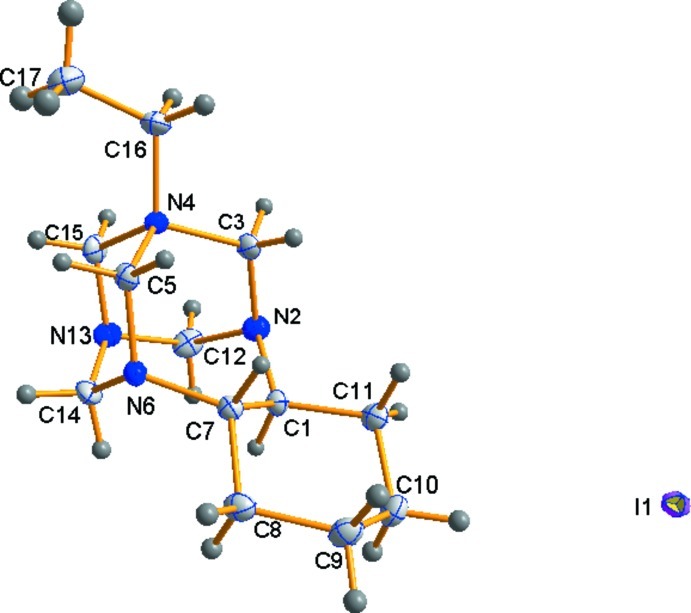
A view of (I) with the numbering scheme. Displacement ellipsoids are drawn at the 50% probability level.

**Fig. 2.**
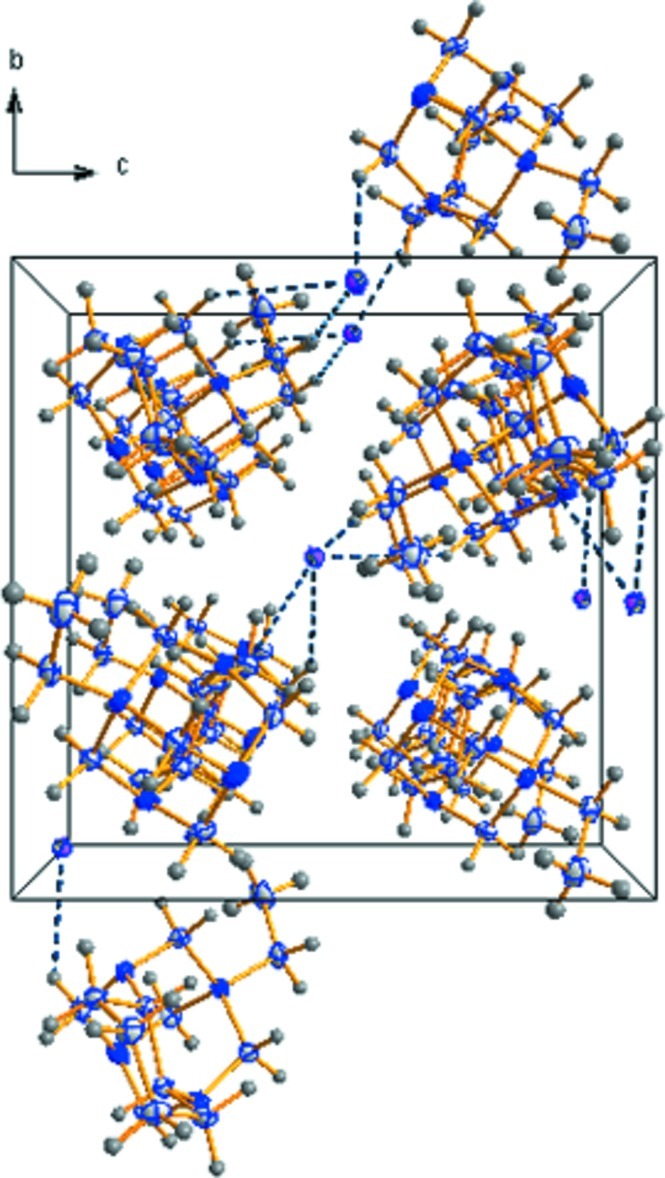
Packing of the ions of the title compound viewed along a axis. C—H···I and C—H···C short contacts are drawn as dashed lines.

## supplementary crystallographic information

### Comment

The aminal (2*S*,7*S*)-1,8,10,12-tetraazatetracyclo [8.3.1.1^8,12^.0^2,7^]pentadecane is interesting because it presents a chiral molecular structure, which contains two pairs of non equivalent nitrogen atoms. (Rivera, Quiroga *et al.*, 2012). Employing the method described, (Rivera *et al.*, 2011*a*) the title compound (I) was readily prepared from (2*S*,7*S*)-1,8,10,12-tetraazatetracyclo [8.3.1.1^8,12^.0^2,7^]pentadecane by alkylation with iodo ethane in dry acetonitrile, at room temperature. The chiral quaternary ammonium salts are used as asymmetric phase-transfer catalysis (Lygo Andrews, 2004) and enantioselective reactions (Kim & Huh, 2001; Park *et al.*, 2004).The molecular structure and atom-numbering scheme for (I) are shown in Fig 1.

The compound (I) crystallizes in the orthorhombic *P*2_1_2_1_2_1_ chiral space group. The asymmetric unit of title molecule, C_13_H_25_N_4_^+^.I^-^, contains 10-ethyl-(2*S*,7*S*)-1,8,10,12-tetraazatetracyclo- [8.3.1.1^8,12^.0^2,7^]pentadecan-10-ium cation and one iodide anion. The *N*-monoalkylation produces ions where one nitrogen atom carries a full positive charge resulting in a distortion of the bond lengths and angles in the heterocyclic ring, compared to the molecular structure in the solid state of hexamethylenetetramine (Becka *et al.*, 1963). The C—N distances and bond angles (C—N—C and N—C—N) vary from 1.426 (3) to 1.575 (3) Å and from 104.97 (15) to 118.54 (16)°, respectively.

Crystallographic data indicate the existence of an anomeric effect (Kakanejadifard & Farnia, 1997; Rivera *et al.*, 2011*b*) in (I), manifest in the following facts: a lengthening of N4—C bond lengths [N4—C3, 1.575 (3) Å; N4—C5, 1.531 (3) Å; N4—C15, 1.529 (3) Å], and shortening of bond lengths N4C—N [C3—N2, 1.432 (3) Å; C5—N6, 1.426 (3) Å; C15—N13, 1.434 (3) Å] comparable with normal bond distances (Allen *et al.*, 1987). Distortion of the C—N—C bond angles decreased the *p* character of non charged N atoms and reduces the N-pyramidality [α (CNC) around N2 = 341.47°; N6 = 342.22°; N13 = 332.7°] as occurring in other aminal derived salts (Rivera *et al.*, 2011*b*,*c*; Rivera, Sadat-Bernal *et al.*, 2012).The geometry of the N—C—C—N moiety is close to the *syn*-periplanar conformation, evidenced by the N2—C1—C7—N6 torsion angle, 37.7 (2)°. The organization of the crystal packing for the title compound exhibits a network connecting ions through C—H···I and C—H···C short contacts (Fig. 2).

### Experimental

*Preparation of 10-ethyl-(2S,7S)-1,8,10,12-tetraazatetracyclo [8.3.1.1^8,12^.0^2,7^] pentadecan-10-ium iodide*

The title compound was synthesized according to the published procedure (Rivera *et al.*, 2011*a*) by reacting (2*S*,7*S*)-1,8,10,12-tetraazatetracyclo[8.3.1.1^8,12^.0^2,7^]pentadecane and iodoethane. After work up a solid was obtained and then dissolved in water. After standing for several days at room temperature, crystals suitable for X-ray diffraction were obtained in 45% yield. Mp = 450–452 K.

### Refinement

All H atoms were discernible in difference Fourier maps and could be refined to reasonable geometry, but according to common practice H atoms bonded to C atoms were kept in ideal positions with C—H = 0.96 Å. *U*_iso_(H) was set to 1.2*U*_eq_(carrier atom). The absolute configuration for chiral centers C1 and C7 was determined using the anomalous dispersion of the iodine site, by refining a Flack parameter (Flack, 1983) based on 1353 Friedel pairs.

### Crystal data

**Table:**

C_13_H_25_N_4_^+^·I^−^	*D*_x_ = 1.625 Mg m^−^^3^
*M**_r_* = 364.3	Melting point: 450 K
Orthorhombic, *P*2_1_2_1_2_1_	Mo *K*α radiation, λ = 0.7107 Å
Hall symbol: P 2ac 2ab	Cell parameters from 3457 reflections
*a* = 10.2227 (5) Å	θ = 3.1–27.0°
*b* = 12.0375 (6) Å	µ = 2.14 mm^−^^1^
*c* = 12.0941 (6) Å	*T* = 120 K
*V* = 1488.25 (13) Å^3^	Prism, colourless
*Z* = 4	0.24 × 0.06 × 0.04 mm
*F*(000) = 736	

### Data collection

**Table:**

Agilent Xcalibur (Atlas, Gemini ultra) diffractometer	3223 independent reflections
Radiation source: Enhance (Mo) X-ray Source	3093 reflections with *I* > 3σ(*I*)
Graphite monochromator	*R*_int_ = 0.016
Detector resolution: 10.3784 pixels mm^-1^	θ_max_ = 27.1°, θ_min_ = 3.1°
ω scans	*h* = −13→9
Absorption correction: multi-scan (*CrysAlis PRO*; Agilent, 2010)	*k* = −14→15
*T*_min_ = 0.746, *T*_max_ = 1	*l* = −10→15
5935 measured reflections	

### Refinement

**Table:**

Refinement on *F*^2^	H-atom parameters constrained
*R*[*F*^2^ > 2σ(*F*^2^)] = 0.017	Weighting scheme based on measured s.u.'s *w* = 1/(σ^2^(*I*) + 0.0004*I*^2^)
*wR*(*F*^2^) = 0.041	(Δ/σ)_max_ = 0.018
*S* = 1.19	Δρ_max_ = 0.23 e Å^−^^3^
3223 reflections	Δρ_min_ = −0.47 e Å^−^^3^
164 parameters	Absolute structure: Flack (1983), 1353 Friedel pairs
0 restraints	Flack parameter: 0.026 (15)
0 constraints	

### Fractional atomic coordinates and isotropic or equivalent isotropic displacement parameters (Å^2^)

**Table:**

	*x*	*y*	*z*	*U*_iso_*/*U*_eq_	
I1	0.995575 (12)	0.462386 (9)	0.966324 (9)	0.01806 (4)	
C1	0.4852 (2)	0.29285 (13)	0.73421 (15)	0.0153 (5)	
N2	0.36266 (17)	0.33495 (15)	0.78149 (16)	0.0157 (5)	
C3	0.2959 (2)	0.26756 (17)	0.86111 (18)	0.0170 (6)	
N4	0.20151 (16)	0.17701 (14)	0.81134 (14)	0.0141 (5)	
C5	0.2742 (2)	0.08504 (16)	0.74916 (17)	0.0146 (6)	
N6	0.35735 (17)	0.12860 (15)	0.66504 (15)	0.0148 (5)	
C7	0.4840 (2)	0.16841 (14)	0.70831 (15)	0.0142 (5)	
C8	0.6008 (2)	0.13825 (18)	0.63737 (19)	0.0198 (7)	
C9	0.7262 (2)	0.16905 (18)	0.6984 (2)	0.0213 (7)	
C10	0.7278 (2)	0.28974 (16)	0.73715 (19)	0.0212 (7)	
C11	0.6056 (2)	0.31806 (19)	0.8036 (2)	0.0180 (6)	
C12	0.2718 (2)	0.37477 (18)	0.69715 (19)	0.0211 (6)	
N13	0.19918 (17)	0.28513 (15)	0.64344 (16)	0.0194 (5)	
C14	0.2834 (2)	0.20109 (17)	0.58957 (18)	0.0191 (6)	
C15	0.1162 (2)	0.23336 (18)	0.72399 (18)	0.0186 (6)	
C16	0.1232 (2)	0.13179 (17)	0.90618 (18)	0.0203 (6)	
C17	0.0267 (2)	0.04169 (19)	0.87726 (19)	0.0289 (7)	
H1c1	0.490912	0.333228	0.66593	0.0183*	
H1c3	0.247872	0.314369	0.91096	0.0204*	
H2c3	0.358654	0.232404	0.908641	0.0204*	
H1c5	0.325356	0.042413	0.80047	0.0175*	
H2c5	0.212023	0.035003	0.716646	0.0175*	
H1c7	0.493127	0.128663	0.776687	0.017*	
H1c8	0.600074	0.059842	0.62289	0.0237*	
H2c8	0.596557	0.178345	0.568841	0.0237*	
H1c9	0.800126	0.155718	0.651276	0.0256*	
H2c9	0.737333	0.120781	0.760916	0.0256*	
H1c10	0.804004	0.302556	0.781695	0.0254*	
H2c10	0.733483	0.338024	0.674158	0.0254*	
H1c11	0.606624	0.395538	0.822422	0.0216*	
H2c11	0.603662	0.274033	0.869799	0.0216*	
H1c12	0.211459	0.426301	0.729818	0.0254*	
H2c12	0.319	0.416333	0.642393	0.0254*	
H1c14	0.23152	0.156343	0.540652	0.0229*	
H2c14	0.342843	0.237356	0.539822	0.0229*	
H1c15	0.062251	0.178719	0.688439	0.0224*	
H2c15	0.062416	0.288707	0.7586	0.0224*	
H1c16	0.078397	0.191556	0.9426	0.0243*	
H2c16	0.181437	0.105459	0.962693	0.0243*	
H1c17	−0.019759	0.019483	0.942586	0.0346*	
H2c17	−0.034168	0.069235	0.823415	0.0346*	
H3c17	0.072673	−0.021042	0.847344	0.0346*	

### Atomic displacement parameters (Å^2^)

**Table:**

	*U*^11^	*U*^22^	*U*^33^	*U*^12^	*U*^13^	*U*^23^
I1	0.01928 (7)	0.01921 (7)	0.01568 (7)	0.00180 (7)	0.00111 (8)	0.00244 (4)
C1	0.0163 (11)	0.0137 (9)	0.0159 (8)	−0.0011 (8)	−0.0006 (11)	−0.0009 (7)
N2	0.0173 (9)	0.0108 (9)	0.0188 (10)	0.0020 (7)	0.0016 (8)	0.0008 (8)
C3	0.0184 (10)	0.0152 (10)	0.0174 (10)	0.0000 (8)	0.0010 (9)	−0.0024 (8)
N4	0.0139 (9)	0.0150 (9)	0.0133 (8)	−0.0001 (7)	0.0012 (8)	0.0007 (7)
C5	0.0180 (10)	0.0118 (10)	0.0140 (10)	−0.0001 (8)	0.0006 (9)	−0.0012 (8)
N6	0.0158 (9)	0.0147 (9)	0.0138 (9)	−0.0025 (7)	−0.0021 (8)	0.0006 (7)
C7	0.0168 (11)	0.0124 (8)	0.0132 (8)	−0.0004 (9)	−0.0006 (10)	0.0005 (7)
C8	0.0216 (11)	0.0155 (11)	0.0222 (12)	−0.0011 (9)	0.0048 (10)	−0.0031 (9)
C9	0.0167 (11)	0.0211 (12)	0.0260 (12)	0.0019 (9)	0.0039 (10)	−0.0022 (10)
C10	0.0165 (11)	0.0212 (12)	0.0257 (12)	−0.0032 (8)	−0.0022 (10)	−0.0010 (10)
C11	0.0203 (11)	0.0150 (11)	0.0187 (11)	−0.0033 (9)	0.0002 (10)	−0.0007 (9)
C12	0.0218 (11)	0.0143 (11)	0.0274 (11)	0.0025 (9)	0.0003 (10)	0.0061 (9)
N13	0.0180 (9)	0.0201 (9)	0.0200 (9)	0.0011 (7)	−0.0001 (8)	0.0059 (7)
C14	0.0218 (11)	0.0234 (11)	0.0121 (10)	−0.0023 (9)	−0.0013 (9)	0.0027 (9)
C15	0.0147 (11)	0.0216 (11)	0.0196 (11)	0.0034 (8)	−0.0044 (9)	0.0028 (9)
C16	0.0206 (11)	0.0254 (12)	0.0148 (10)	−0.0005 (9)	0.0045 (9)	0.0005 (9)
C17	0.0255 (12)	0.0346 (12)	0.0265 (11)	−0.0074 (11)	0.0048 (10)	0.0037 (9)

### Geometric parameters (Å, º)

**Table:**

C1—C7	1.530 (2)	C9—H2c9	0.96
C1—C11	1.521 (3)	C10—C9	1.527 (3)
C1—H1c1	0.96	C10—C11	1.524 (3)
N2—C1	1.467 (3)	C10—H1c10	0.96
N2—C3	1.432 (3)	C10—H2c10	0.96
N2—C12	1.460 (3)	C11—H1c11	0.96
C3—H1c3	0.96	C11—H2c11	0.96
C3—H2c3	0.96	C12—H1c12	0.96
N4—C3	1.575 (3)	C12—H2c12	0.96
N4—C5	1.531 (3)	N13—C12	1.462 (3)
N4—C15	1.529 (3)	N13—C14	1.480 (3)
N4—C16	1.501 (3)	N13—C15	1.434 (3)
C5—H1c5	0.96	C14—H1c14	0.96
C5—H2c5	0.96	C14—H2c14	0.96
N6—C5	1.426 (3)	C15—H1c15	0.96
N6—C7	1.476 (3)	C15—H2c15	0.96
N6—C14	1.472 (3)	C16—H1c16	0.96
C7—H1c7	0.96	C16—H2c16	0.96
C8—C7	1.514 (3)	C17—C16	1.507 (3)
C8—C9	1.526 (3)	C17—H1c17	0.96
C8—H1c8	0.96	C17—H2c17	0.96
C8—H2c8	0.96	C17—H3c17	0.96
C9—H1c9	0.96		
			
N2—C1—C7	114.27 (17)	H1c9—C9—H2c9	105.87
N2—C1—C11	114.02 (16)	C9—C10—H1c10	109.47
N2—C1—H1c1	102.26	C9—C10—H2c10	109.47
C7—C1—C11	108.35 (17)	C11—C10—C9	111.49 (18)
C7—C1—H1c1	108.66	C11—C10—H1c10	109.47
C11—C1—H1c1	108.95	C11—C10—H2c10	109.47
C1—N2—C3	118.26 (16)	H1c10—C10—H2c10	107.37
C1—N2—C12	112.59 (17)	C1—C11—C10	109.11 (18)
C12—N2—C3	110.62 (17)	C1—C11—H1c11	109.47
N2—C3—N4	115.28 (17)	C1—C11—H2c11	109.47
N2—C3—H1c3	109.47	C10—C11—H1c11	109.47
N2—C3—H2c3	109.47	C10—C11—H2c11	109.47
N4—C3—H1c3	109.47	H1c11—C11—H2c11	109.83
N4—C3—H2c3	109.47	N2—C12—N13	113.02 (17)
H1c3—C3—H2c3	102.97	N2—C12—H1c12	109.47
C3—N4—C5	113.01 (15)	N2—C12—H2c12	109.47
C3—N4—C15	107.85 (15)	N13—C12—H1c12	109.47
C3—N4—C16	106.58 (15)	N13—C12—H2c12	109.47
C5—N4—C15	104.97 (15)	H1c12—C12—H2c12	105.67
C16—N4—C5	111.84 (15)	C12—N13—C14	113.87 (17)
C16—N4—C15	112.64 (15)	C12—N13—C15	108.62 (17)
N4—C5—H1c5	109.47	C14—N13—C15	110.25 (17)
N4—C5—H2c5	109.47	N6—C14—N13	115.54 (18)
N6—C5—N4	111.96 (16)	N6—C14—H1c14	109.47
N6—C5—H1c5	109.47	N6—C14—H2c14	109.47
N6—C5—H2c5	109.47	N13—C14—H1c14	109.47
H1c5—C5—H2c5	106.87	N13—C14—H2c14	109.47
C7—N6—C5	112.91 (16)	H1c14—C14—H2c14	102.63
C7—N6—C14	118.54 (16)	N4—C15—H1c15	109.47
C14—N6—C5	110.77 (16)	N4—C15—H2c15	109.47
C1—C7—H1c7	108.1	N13—C15—N4	108.96 (17)
N6—C7—C1	113.40 (17)	N13—C15—H1c15	109.47
N6—C7—C8	114.37 (16)	N13—C15—H2c15	109.47
N6—C7—H1c7	103.22	H1c15—C15—H2c15	109.98
C8—C7—C1	110.15 (17)	N4—C16—C17	115.63 (18)
C8—C7—H1c7	106.98	N4—C16—H1c16	109.47
C7—C8—C9	109.28 (18)	N4—C16—H2c16	109.47
C7—C8—H1c8	109.47	C17—C16—H1c16	109.47
C7—C8—H2c8	109.47	C17—C16—H2c16	109.47
C9—C8—H1c8	109.47	H1c16—C16—H2c16	102.52
C9—C8—H2c8	109.47	C16—C17—H1c17	109.47
H1c8—C8—H2c8	109.66	C16—C17—H2c17	109.47
C8—C9—C10	112.85 (18)	C16—C17—H3c17	109.47
C8—C9—H1c9	109.47	H1c17—C17—H2c17	109.47
C8—C9—H2c9	109.47	H1c17—C17—H3c17	109.47
C10—C9—H1c9	109.47	H2c17—C17—H3c17	109.47
C10—C9—H2c9	109.47		
